# Roles of Cyclic AMP Response Element Binding Activation in the ERK1/2 and p38 MAPK Signalling Pathway in Central Nervous System, Cardiovascular System, Osteoclast Differentiation and Mucin and Cytokine Production

**DOI:** 10.3390/ijms20061346

**Published:** 2019-03-17

**Authors:** Yasuhiko Koga, Hiroaki Tsurumaki, Haruka Aoki-Saito, Makiko Sato, Masakiyo Yatomi, Kazutaka Takehara, Takeshi Hisada

**Affiliations:** 1 Department of Allergy and Respiratory Medicine, Gunma University Graduate School of Medicine, 3-39-15 sho-wa machi Maebashi, Gunma 371-8511, Japan; m12702056@gunma-u.ac.jp (H.T.); a-haruka@gunma-u.ac.jp (H. A-S.); m1620012@gunma-u.ac.jp (M.S.); m09702007@gunma-u.ac.jp (M.Y.); k-kanplude3@jcom.home.ne.jp (K.T.); 2 Gunma University Graduate School of Health Sciences, 3-39-22 sho-wa machi Maebashi, Gunma 371-8514, Japan; hisadat@gunma-u.ac.jp

**Keywords:** ERK1/2, p38 MAPK, CREB, periostin, osteoclast differentiation, GM-CSF, mucin, MUC5AC, migration, steroidogenesis

## Abstract

There are many downstream targets of mitogen-activated protein kinase (MAPK) signalling that are involved in neuronal development, cellular differentiation, cell migration, cancer, cardiovascular dysfunction and inflammation via their functions in promoting apoptosis and cell motility and regulating various cytokines. It has been reported that cyclic AMP response element-binding protein (CREB) is phosphorylated and activated by cyclic AMP signalling and calcium/calmodulin kinase. Recent evidence also points to CREB phosphorylation by the MAPK signalling pathway. However, the specific roles of CREB phosphorylation in MAPK signalling have not yet been reviewed in detail. Here, we describe the recent advances in the study of this MAPK-CREB signalling axis in human diseases. Overall, the crosstalk between extracellular signal-related kinase (ERK) 1/2 and p38 MAPK signalling has been shown to regulate various physiological functions, including central nervous system, cardiac fibrosis, alcoholic cardiac fibrosis, osteoclast differentiation, mucin production in the airway, vascular smooth muscle cell migration, steroidogenesis and asthmatic inflammation. In this review, we focus on ERK1/2 and/or p38 MAPK-dependent CREB activation associated with various diseases to provide insights for basic and clinical researchers.

## 1. Introduction

Three mitogen-activated protein kinase (MAPK) signalling pathways, including extracellular signal-related kinase (ERK) 1/2, p38 MAPK and c-Jun N-terminal kinase (JNK), play significant roles in the inflammatory response involved in various human diseases. Among the many substrates of MAPK, cyclic adenosine monophosphate (cAMP) response element-binding protein (CREB) is a lesser-known critical player in inflammatory diseases associated with MAPK signalling. CREB was originally reported as a mediator of the cAMP signalling pathway [1]. Binding of cAMP to the regulatory subunit of protein kinase A (PKA) results in the dissociation and translocation of the catalytic subunit of PKA to directly phosphorylate CREB. CREB phosphorylation is also dephosphorylated by protein phosphatase 1 and protein phosphatase 2B (calcineurin) [2]. Thereafter, other kinases including protein kinase C, calcium/calmodulin-dependent protein kinases (CaMKs), ERK1/2 and p38 MAPK were found to activate CREB [3,4,5,6]. Originally, Xing et al. reported that nerve growth factor (NGF) activates CREB at the Ser133 residue through ERK1/2 and p38 MAPK phosphorylation in PC12 cells [7]. Direct phosphorylation of CREB by ERK1/2 or p38 MAPK has not been reported. The ERK1/2 or p38 MAPK pathway for CREB phosphorylation occurs in part through an indirect pathway mediated by pp90 ribosomal S6 kinase (RSK), mitogen- and stress-activated protein kinase (MSK)1/2, MAPKAP kinase 2 phosphorylation [8]. Regulatory genes by ERK/p38 MAPK or cAMP/PKA are varied and depend on the agonist stimulation. There is no review focusing on CREB activation in the MAPK signalling pathway. Therefore, in this review, we describe the crosstalk between ERK1/2 and p38 MAPK signal transduction that results in neuronal development, cardiac fibrosis through periostin production, osteoblast differentiation, vascular smooth muscle cell (VSMC) migration and airway inflammation by mucin production and granulocyte-macrophage colony-stimulating factor (GM-CSF) secretion for CREB activation.

## 2. Differential Roles of PKA, CaMKIV and ERK/p38 MAPK Axis in CREB Activation in the Central Nervous System

CREB plays pivotal roles in learning, long-term memory and synaptic plasticity. PKA, CaMKIV, ERK and p38 MAPK are known to regulate CREB phosphorylation in neurons. Physiological roles of various kinase-dependent CREB phosphorylation has been investigated previously [8].

High levels of cAMP are linked to the extensive axonal growth during development in the embryonic central nervous system [9]. cAMP is important for stabilizing of growth cones in the developing nervous system. Increasing neuronal cAMP can develop neurite growth and regeneration [10]. cAMP directly activates PKA and exchange protein directly activated by cAMP (EPAC) and PKA leads to CREB phosphorylation, resulting in axon growth [11]. Both PKA and Epac mediate cAMP-induced neurite extension in PC12 cells [12] and axonal regeneration in neurons [13]. PKA but not Epac, facilitates cAMP-induced neuronal differentiation via CREB phosphorylation [14].

Another CREB kinase, CaMKIV, phosphorylates CREB in vitro [15]. Activity-dependent CaMKIV-CREB activation plays a vital role in the consolidation of long-term memory revealed in dominant negative CaMKIV transgenic mice [16]. CaMKIV but not the PKA or MAPK pathway, is essential for establishing the late phase of cerebellar long-term depression mediated by CREB phosphorylation [17].

ERK/p38 MAPK induced CREB phosphorylation distinct from the cAMP/PKA pathway in neuronal cells has been investigated. Xing et al. reported that nerve growth factor (NGF) activates ERK, which in turn activates RSK. NGF also activates p38 MAPK and its downstream effector, MAPK-activated protein kinase 2 (MAPKAP kinase 2), resulting in CREB phosphorylation in PC12 cells. Furthermore, the ERK/RSK and p38 MAPK/MAPKAP kinase 2 pathways co-ordinately contribute to CREB phosphorylation in NGF-treated PC12 cells because suppression of these pathways is necessary for completely inhibiting NGF-induced CREB phosphorylation. Retinoic acid is a potent regulator of neuronal cell differentiation and induces ERK1/2 phosphorylation, which results in CREB phosphorylation in PKA-deficient PC12 cells and primary neuronal cells via a PKA-independent pathway [18].

Crosstalk between PKA and ERK associated with the formation of long-lasting neuronal plasticity has been reported in neuronal cells. Toropomyosin receptor kinases (Trk) is important for neurite outgrowth. Trk signalling leads to activation of the cAMP-ERK pathway, which promotes increased neurite outgrowth and regeneration in isolated cerebellar [19]. Ca^2+^ induces ERK-CREB phosphorylation through cAMP/PKA activation in PC12 cells and hippocampal neurons. RSK2 has been identified as Ca^2+^-activated CREB kinase in PC12 cells and hippocampal neurons [20]. Activation of the PKA pathway by forskolin or stimulation of D1-like dopamine receptors induces intracellular Ca^2+^ release. Intracellular Ca^2+^ release activates the ERK/RSK pathway mediated by PKC, Rap1-B-Raf and PYK2 complex and then phosphorylates CREB in striatal neurons [21]. Furthermore, C-fibre activation of multiple metabotropic, ionotropic receptors results in ERK-CREB phosphorylation mediated by PKC and PKA pathways in dorsal horn neurons associated with plasticity in the spinal cord. The activated ERK-CREB pathway contributes to the acute phase of central sensitization, leading to long-lasting changes in sensory processing [22].

In summary, it is known that PKA, CaMKIV, ERK and p38 MAPK can phosphorylate CREB upon stress and in the presence of neuronal growth factors and excitatory neurotransmitters. While PKA and CaMKIV phosphorylate CREB directly, ERK and p38 MAPK phosphorylate CREB indirectly in a process mediated by RSK and MAPKAP kinase 2, respectively. These pathways co-ordinately contribute to the development of the central nervous system mediated CREB phosphorylation.

## 3. Roles of ERK1/2 and p38 MAPK in Periostin Production in Cardiovascular Disease

### 3.1. Alcoholic Cardiomyopathy

Alcoholic cardiomyopathy (ACM) is diagnosed by linking the dilation and impaired contraction of one or both myocardial ventricles with a history of heavy alcohol consumption [23]. Several molecular mechanisms underlie the adverse effects of alcohol, including apoptotic cell death, oxidative stress, derangements in fatty acid metabolism and transport, impaired mitochondrial bioenergetics/stress and accelerated protein catabolism [24]. Ang II contributes to alcohol-induced cardiac dysfunction and downregulating Ang II could improve ACM [25,26]. Alcohol is metabolized into acetaldehyde (ACA) in the liver by alcohol dehydrogenase (ADH). Moreover, ACA is metabolized by acetaldehyde dehydrogenase (ALDH) into acetic acid [27]. ALDH2 has been identified as a crucial cardioprotective enzyme, which enables a remarkable reduction in cardiac injury after ischemic/reperfusion events [24]. Doser et al. reported that ALDH2 transgenic mice, rescued from alcohol-induced contractile dysfunction and cardiac hypertrophy by the inhibition of CREB activation [28]. Liu et al. demonstrated that Alda-1, an activator of ALDH2, alleviated alcohol-induced cardiac dysfunction. It also decreased angiotensinogen and Ang II levels in vitro in cardiomyocytes and in mouse hearts by suppressing p38 MAPK/CREB activation [29]. This suggests a new target for the treatment of ACM [27,30].

### 3.2. Cardiac Remodeling

Cardiac remodelling is defined as changes in the heart structure due to various pathologic events. Cardiac remodelling is a risk factor for chronic heart failure due to it causing reduced contractility and high mortality [31]. The extracellular cardiac matrix (ECM) is a dynamic support structure that is remodelled following cardiac injury and heart failure. Progressive ECM remodelling is closely linked to heart failure severity and poor prognosis [32]. Recent studies in a cardiac pressure-overloading mouse model and in patients with hypertension because of aortic constriction suggest that heart failure-associated alteration in cardiac ECM is associated with activation of the local renin–angiotensin system (RAS) [33,34]. Furthermore, RAS activation plays a significant role in cardiac remodelling [35]. Previous studies suggest that cardiac RAS activation in cardiac remodelling is induced by an overload in heart pressure. Angiotensin II (Ang II) receptor blockers or angiotensin-converting enzyme inhibitors are effective in ameliorating heart failure, including alleviating ECM remodelling and preventing cardiac remodelling [32]. Therefore, Ang II induces excessive ECM deposition.

**Figure 1:**
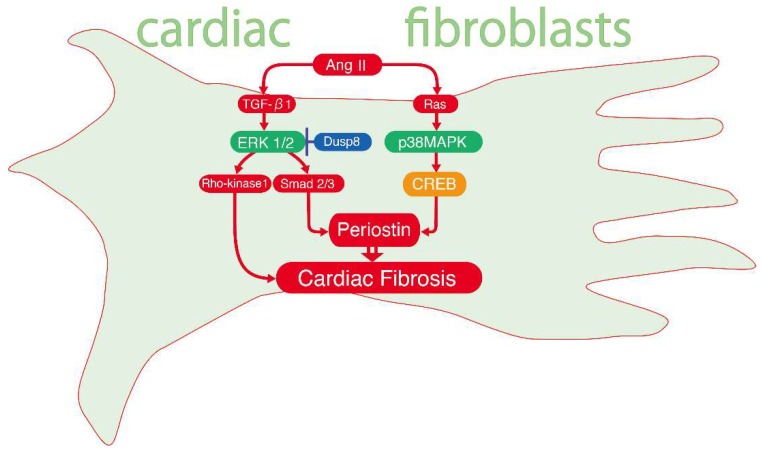
Ang-II induced cardiac fibrosis mediated by periostin. Ang II activates TGF-β1 and Ras, thus inducing ERK1/2 and p38 MAPK phosphorylation, respectively. ERK1/2 stimulates Smad2/3, which is suppressed by Dusp8 and p38 MAPK induced cAMP response-element binding protein (CREB) activation then periostin, produced in local cardiac fibroblasts following cardiac fibrosis.

It has been demonstrated that Ang II induces the production of periostin [36], a matricellular protein, enabling it to bind both to cellular receptors and the ECM. Periostin, a key regulator of cardiac fibrosis, is secreted primarily from osteoblasts and fibroblasts and expressed in bones, kidneys, lungs and heart valves in adult mammals [37]. Periostin expression is significant during remodelling in mouse hearts [38] and in human failing hearts [39]. Periostin overexpression in rat heart leads to cardiac dysfunction, with significantly increased fibrosis [40]. However, periostin-knockout mice show less fibrosis after long-term pressure overload [41]. Li et al. showed that Ang II enhances periostin expression in a rat model and in cultured rat cardiac fibroblasts. They demonstrated that alternative signalling cascades for periostin production were induced by the p38 MAPK and ERK1/2 pathway. RasGRP1, Ras and p38 MAPK are signalling molecules that mediate Ang II-induced CREB activation for periostin production. ERK1/2 also participates in Ang II-induced periostin expression by regulating transforming growth factor (TGF)-β1/Smad2/3 pathway, distinct from the p38 MAPK-CREB pathway [42]. The upregulated periostin expression promotes angiogenesis by activating ERK1/2 and FAK signalling pathways and increasing the secretion of VEGF and Ang I [43]. In myocardial infarction model rats, ERK1/2 phosphorylation mediates TGF-β1-induced cardiac fibrosis via Rho-kinase 1 activation but not the JNK or p38 MAPK pathway [44]. ERK1/2 also mediates cigarette smoke extract-induced periostin expression in pulmonary arterial smooth muscle cells [45]. In a dilated cardiomyopathy mouse model overexpressing the Fas ligand (an inducer of apoptosis via caspase 3 activation), Fas ligand-activated periostin expression is mediated by the ERK1/2 pathway [46,47]. The dual-specificity phosphatases (DUSPs) are regulators of the basic condition and duration of MAPK signalling. Liu et al. reported that the *DUSP8* gene inhibits cardiac ventricular remodelling, thereby suppressing ERK1/2 activity [48]. These studies have revealed that ERK1/2 and p38 MAPK coordinate to regulate periostin expression in cardiac fibrotic disease (Figure 1).

## 4. Crosstalk between ERK1/2 and CREB-p38 MAPK Signalling in Osteoclast Differentiation

Osteoclasts are differentiated from the monocyte/macrophage lineage of hematopoietic cells. Bone homeostasis is regulated by bone formation and bone resorption activity. Osteoclasts that are responsible for bone resorption are involved in bone homeostasis together with osteoblasts, which constitute the bone matrix [49,50,51]. Osteoclast differentiation is controlled by cytokines, including a receptor activator of nuclear factor kappa B (NF-κB), ligand (RANKL) and macrophage colony-stimulating factor (M-CSF) [52]. The binding of M-CSF to its receptor results in the activation of MAPK and Akt (a serine/threonine-specific protein kinase) cascades for osteoclast cell survival. RANKL stimulation results in the activation of downstream signalling via NF-κB, ERK1/2, p38 MAPK and Akt to induce the expression of genes that are crucially essential to promote osteoclast differentiation, including c-Fos and nuclear factor of activated T-cells, cytoplasmic 1 (NFATc1) [53,54], which are the master transcription factors for osteoclastogenesis [55,56].

ERK activation is central for the survival of mature osteoclasts [57] and stable expression of c-Fos induces the expression of NFATc1 following M-CSF stimulation in bone marrow-derived macrophages [55,58]. Integrins mediate intracellular signalling upon agonist stimulation and α_V_β_3_ integrin is expressed in osteoclasts. α_V_β_3_ integrin with c-Fms (CSF-1 receptor) collaboratively mediates osteoclast differentiation through the ERK1/2 and c-Fos signalling pathway with M-CSF stimulation. Interleukin (IL)-1α also promotes ERK activation for the survival of osteoclasts by preventing their apoptosis [59]. A recent study has revealed that the p38 MAPK-CREB pathway plays a significant role in the RANKL-mediated osteoclast differentiation. CREB is essential to induce the transcription of both *c-Fos* and *NFATc1* during osteoclast differentiation through B-cell adaptor for phosphatidylinositol 3-kinase (Pl3K) (BCAP) or Ameloblastin (Ambn) [60,61]. BCAP activates CREB phosphorylation in bone marrow-derived monocyte/macrophage lineage cells under TNF-α or RANKL stimulation in osteoclast differentiation. BCAP overexpression increased and BCAP knockdown by siRNA reduced, TNF-α or RANKL-induced osteoclast differentiation by regulating both c-Fos and NFATc expressions via CREB phosphorylation. TNF-α or RANKL-induced CREB phosphorylation inhibited by p38 MAPK inhibitor, SB203580 and overexpression of BCAP enhances TNF-α or RANKL-induced CREB phosphorylation together with c-Fos and NFATc1 expression, indicating that CREB is essential for inducing c-Fos and NFATc1 upon TNF-α or RANKL stimulation mediated by BCAP [60]. Ambn is an extracellular matrix protein that is mainly associated with tooth development. Ambn also suppresses osteoclast differentiation by inhibiting RANKL expression [62]. A recent study showed that Ambn suppresses RANKL-induced osteoclast differentiation by inhibiting p38 MAPK-CREB phosphorylation and downregulating c-Fos-NFATc1 axis [61]. These results indicate that p38 MAPK-CREB phosphorylation is important for RANKL-induced c-Fos-NFATc1 axis via Ambn. Sato et al. have shown that CaMK IV activates downstream pathways that are mediated by CREB. The CaMK IV/CREB pathway is essential for RANKL-induced *c-Fos* and *NFATc1* activation. Pharmacological inhibition of CaMK IV, as well as the genetic ablation of CaMK IV, reduce CREB phosphorylation and c-Fos expression [63]. Although the dominance of CaMKIV or p38 MAPK in CREB phosphorylation in osteoclast differentiation has not been reported, Wu et al. found the convergence of a fast CaMKIV-CREB pathway and a slow ERK-CREB pathway under physiologic synaptic stimulation in neuron cells [64]. Therefore, CaMKIV- and p38 MAPK-induced CREB phosphorylation may occur at different times in osteoclast differentiation.

p38 MAPK is activated following MAPK kinase kinase 6 (MKK6) activation [65] upon RANKL stimulation. Receptor for activated C kinase 1 (RACK1), a scaffold protein linked with TNF receptor-associated factor 6 (TRAF6), promotes MKK6-p38 MAPK signalling in response to RANKL and is involved in osteoclast differentiation [66]. Bisphosphonates have been used for the treatment of osteoporosis. Nitrogen-containing bisphosphonates, minodronate and alendronate, inhibit RANKL and M-CSF induced osteoclast formation by the suppression of ERK1/2 and Akt activation [67].

**Figure 2:**
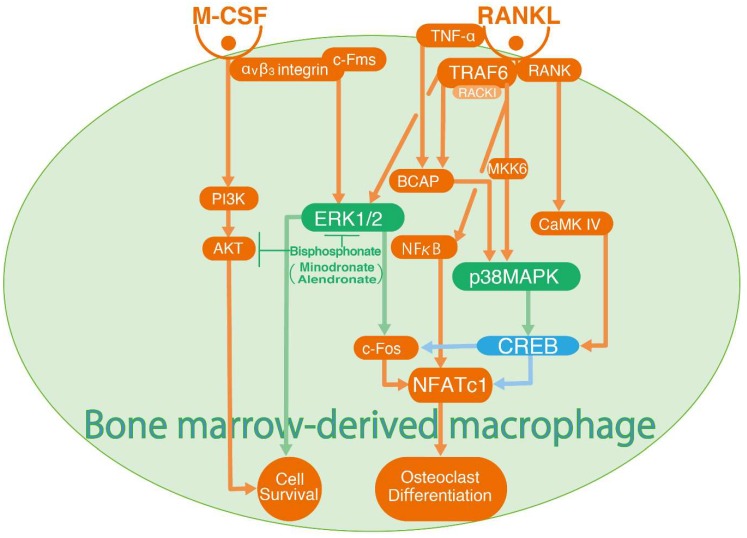
Regulatory mechanisms of macrophage-colony stimulating factor (M-CSF) and receptor activator of nuclear factor kappa B, ligand (RANKL) induced osteoblast differentiation and survival. ERK1/2, p38 MAPK, BCAP, NFκB and CaMK IV are functioning downstream of the RANKL complex. While ERK1/2 induces cell survival and osteoclast differentiation under M-CSF and RANKL complex stimulation, p38 MAPK and CaMK IV activate c-Fos and NFATc1 for osteoclast differentiation mediated by CREB phosphorylation.

Taken together, the ERK1/2 and p38 MAPK-CREB pathways play important roles in bone homeostasis. Although the underlying mechanism and the roles of CREB activation in osteoclast differentiation have not been fully elucidated, understanding of the ERK1/2 and p38 MAPK-CREB pathways will help develop therapeutic strategies for various bone diseases (Figure 2). 

## 5. Mucin Production and MAPK Signalling

Lung mucus is secreted by lung epithelial cells and plays a pivotal role in airway clearance and immunity in healthy lungs [68]. Lung mucus is produced excessively in lung diseases, such as bronchial asthma and chronic obstructive pulmonary disease (COPD) and primary lung carcinomas [69,70]. Airway inflammation is a major trigger of mucin (MUC) gene expression and mucus secretion. MUC5AC and MUC5B are the secretory gel-forming mucins in lung mucus and in secretions from normal airways. MUC5AC is produced excessively in bronchial asthma and COPD. However, MUC5B production at airway surfaces decreases by as much as 90% in many patients. Unlike MUC5, MUC4 is a member of the transmembrane mucin family that is expressed in airway epithelial cells. Abnormal expression of MUC4 has been reported in primary lung carcinomas [71].

Many stimuli, such as cytokines, epidermal growth factor receptor (EGFR) ligands, microorganisms and cigarette smoke induce MUC5 production by the activation of several signalling pathways and transcriptional factors. The pathways for MUC5 production are mainly through four cascades, EGFR signalling, cytokine signalling, toll-like receptor (TLR) signalling and reactive oxygen species (ROS) signalling. In the signalling pathways for MUC5 production, MAPK are the key molecules, except for the pathway through which IL-13 induces MUC5 production. MAPK signalling pathways result in the activation of several alternative transcription factors required for MUC5AC upregulation. Each stimulus activates a different MAPK signalling pathway.

TGF-α and amphiregulin (a widely expressed transmembrane tyrosine kinase) bind EGFR and activate Ras or Raf and they in turn phosphorylate ERK1/2. ERK1/2 and activate transcriptional factors, such as activator protein (AP) 1, specificity protein (SP) 1 and CREB following the transcription of MUC5AC. Perrais et al. reported that the transcription factor SP1 is essential for EGF and TGF-α-mediated *MUC5AC* up-regulation [72,73].

Several cytokines including IL-1β, tumour necrosis factor (TNF)-α and IL-13 induce MUC5AC production [74]. Song et al. reported that ERK and p38 MAPK but not JNK signalling, are essential for IL-1β and TNF-α-induced *MUC5AC* gene expression. Furthermore, the activation of MSK 1 and CREB are crucial aspects of the intracellular mechanisms that mediate *MUC5AC* gene expression [75]. This study also showed that CRE in the *MUC5AC* promoter might play an essential role in these processes by binding to CREB. LPA, LPS or TNF-α stimulation induces the production of cytokines, such as IL-8 and IL-1β, which is mediated by CREB phosphorylation. ERK1/2 or p38 MAPK facilitates MSK phosphorylation directly and thus phosphorylates CREB under these agonists [76]. Because the ERK or p38 MAPK inhibitor equally inhibited both IL-1β- or TNF-α-induced MUC5AC mRNA expression and CREB phosphorylation in normal human nasal epithelial cells [75], the difference between ERK1/2 and p38 MAPK in IL-1β or TNF-α-induced MUC5AC production mediated by MSK-CREB phosphorylation does not appear to be significant. On the other hand, STAT 6 phosphorylation but not MAPK signalling, is essential for IL-13-induced MUC5AC production. IL-13, which is a central mediator of airway remodelling in asthma [77], increases MUC5AC expression by indirect mechanisms, including STAT6 phosphorylation and suppression of the transcription factor, forkhead box protein A2 (FOXA2) [78,79]. Microbial components, including peptidoglycan, lipopolysaccharide, flagellin and nucleotides bind to TLR and induce myeloid differentiation primary response: MyD 88 and TRAF activation following the phosphorylation of MKK 3/6 and p38 MAPK. TLR signals induce AP-1 activation following the transcription of *MUC5AC* [80,81,82,83,84]. Cigarette smoke stimulates nicotinamide adenine dinucleotide phosphate (NADPH) oxidase following the generation of intracellular ROS [85]. ROS stimulate the transcription of *MUC5AC* through the activation of two signalling pathways. The first involves the activation of amphiregulin and EGFR. The amphiregulin binds EGFR and Ras or Raf is activated. MEK is phosphorylated following the phosphorylation of ERK1/2. The second involves ROS activation of Src and subsequently JNK. Both pathways activate Jun D and Fos-related antigen 2 (Fra-2) and thereby stimulate transcription of *MUC5AC* through AP1 [86].

Abnormal expression of MUC4 has been reported in non-small lung carcinoma and especially in adenocarcinomas. MUC4 is associated with male smokers, solid adenocarcinomas, negative TTF-1 expression, wild-type *EGFR*, HER2 protein expression and poorer prognoses [87]. In small-sized lung adenocarcinomas, high MUC4 expression correlated with a short disease-free interval and a poor survival rate [88]. Diesel exhaust particles (DEPs), the major contributors to air pollution, significantly increased the expression of MUC4. MUC4 expression was inhibited by pre-treatment with p38 MAPK and CREB inhibitors in NCI-H292 (ATCC^®^ CRL-1848^TM^) and primary nasal epithelial cells stimulated with DEPs [89].

**Figure 3:**
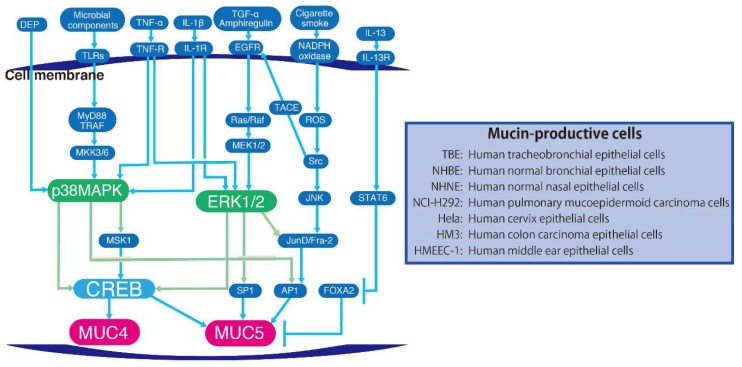
Regulation of the mucus production by MAPK signalling. Multiple stimuli induce phosphorylation of MAPKs following activation of transcriptional factors and production of mucus. IL-13 increases MUC5AC expression by indirect mechanisms including STAT6 phosphorylation through FOXA2 suppression.

Collectively, MAPKs are the key molecules in the signalling pathways to produce MUC5. While p38 MAPK is essential for mucin production induced by IL-1β, TNF-α, the microbial components and DEPs via CREB phosphorylation, ERK1/2 is associated with TGF-α, IL-1β, TNF-α and cigarette-induced mucin production. JNK is activated by intracellular ROS induced by cigarette smoke. However, IL-13-induced mucin production is not related to MAPK signalling but with STAT6 phosphorylation (Figure 3).

## 6. Roles of ERK1/2 and p38 MAPK-CREB Signalling in Vascular Smooth Muscle Cell Migration

Accumulation of vascular smooth muscle cell (VSMC) in the intima of arteries plays a key role in atherogenesis [90]. Upon endothelial injury, VSMC migrates into the intima, resulting in pro-inflammatory cytokine production, such as IL-6 causing vascular inflammation. VSMC migration also plays a central role in atherogenesis [91]. The relative enzymatic activity of myosin light chain kinase and myosin light chain phosphatase (MLCP) is the main regulatory mechanism of VSMC migration. MLCP activity is regulated by Rho-kinase and inhibitory phosphoprotein of muscle myosin phosphatase (CPI-17), decreasing phosphatase activity [92,93]; while a myosin phosphatase-Rho interacting protein, p116^Rip^ increases MLCP activity by binding to myosin, actin and myosin phosphatase target subunit 1 [94,95].

Other regulatory mechanisms of VSMC migration have been investigated. Ang II has various roles in regulating VSMC growth, apoptosis and migration. An earlier study has shown that Ang II activates ERK1/2 and JNK in VSMC [96]. Exendin-1, a glucagon-like peptide-1 receptor agonist, used for type-2 diabetes mellitus treatment, attenuates the ERK1/2 and JNK pathway, resulting in Ang II-induced VSMC migration and proliferation [97]. Sitagliptin, a DPP-4 inhibitor, also inhibits high glucose-induced VSMC migration by suppressing ERK1/2 signalling [98]. Advanced glycation end products (AGEs), which is produced by the Maillard reaction following the persistent exposure to high blood glucose, stimulates ERK1/2 and p38 MAPK but not JNK activity, facilitating VSMC migration [99]. PDGF-BB also induces VSMC migration and proliferation through ROS-mediated ERK1/2 and p38 MAPK activation. Paeoniflorin, an herbal constituent often used in traditional Chinese medicine, improves myocardial infarction through an anti-inflammatory function, inhibits PDGF-BB-induced ERK1/2 and p38 MAPK activation [100,101]. Chronic hypoxia is a factor for the induction of VSMC migration and proliferation via TGF β1 and ERK1/2 activation. C1q-TNF-related protein-9 (CTRP9), having structural homology to adiponectin and anti-fibrotic effects, regulates hypoxia-induced VSMC migration and proliferation by suppressing ERK1/2 activation [102]. 

CREB activation has also been demonstrated to be important for VSMC migration. Ono et al. reported that TNF-α-induced VSMC migration is mediated by CREB activation via the p38 MAPK pathway. p38 MAPK inhibitor abolishes both TNF-α-induced both CREB phosphorylation and VSMC migration, while MEK, JNK, PI3-kinase or PKA inhibitor does not inhibit TNF-α-induced CREB phosphorylation. Transfection of adenovirus vector expressing dominant-negative of CREB results significantly inhibited TNF-α-induced VSMC migration and RAC1 protein expression, indicating that CREB activation mediates TNF-α-induced VSMC migration. The reaction is mediated by RAC1, a Rho family small GTPase [103]. UTP, one of the extracellular nucleotides, is released from cardiomyocytes during myocardial infarction [104] and the UTP-induced ERK1/2 and p38 MAPK pathway regulate VSMC migration and osteopontin expression via CREB activation [105]. Polyunsaturated fatty acids such as arachidonic acid resulted in lipoxygenase formation and further converted into hydroxyeicosatetraenoic acids (HETEs). It has been reported that two HETEs are involved in CREB mediated VSMC migration. While 12(S)-HETE activate Src and p38 MAPK-CREB pathway, 15(S)-HETE induce ERK1/2, p38 MAPK and JNK-CREB pathway. Both HETEs have atherogenic effects on IL-6 production and VSMC migration via MAPK-CREB activation [106,107]. Among various agonists, PDGF-BB, AGEs and 15(S)-HETE have been shown to activate both ERK1/2 and p38 MAPK for VSMC migration.

## 7. Regulatory Mechanism of GM-CSF Secretion by cAMP and the EKR1/2-p38 MAPK Signalling Pathway

Granulocyte macrophage-CSF (GM-CSF) is important for the process of maturation of macrophages and granulocytes [108,109]. GM-CSF can be produced by, and acts on, various cell types the expression of which is known to be increased in numerous respiratory diseases including bronchial asthma [110]. Gene transfer of GM-CSF to rat lungs induces eosinophilia, monocytosis and fibrotic reactions in the airway [111,112]. GM-CSF plays a role in local respiratory inflammation and regulating the growth, proliferation and maintenance of neutrophils, macrophages and eosinophils [113]. GM-CSF is a 23-kD glycoprotein and released from airway constituted cells, bronchial epithelial cells, pulmonary fibroblasts and airway smooth muscle cells [114,115,116]. GM-CSF is secreted upon stimulation with agents and cytokines including TNF-α, IL-1β, IL-4, IL-13, viruses and histamine [117,118,119]. GM-CSF is produced by p38 MAPK activation after TNF-α, IL-1α and platelet activating factor (PAF) stimulation in human bronchial epithelial cells [120]. In this pathway, MKK3 and MKK6 are the upstream regulators of p38 MAPK. In human bronchial epithelial cells, *Chlamydophila pneumonia* antigen also produces GM-CSF via p38 MAPK but without JNK, ERK1/2 or PI-3K activity [121]. LPS induces MKK-1 phosphorylation and both ERK1/2 and p38 MAPK phosphorylation are required for GM-CSF secretion [122]. Though p38 MAPK plays crucial role for TNF-α induced GM-CSF secretion, compared to both ERK1/2 and JNK, ERK1/2 but not p38 MAPK is important for GM-CSF mRNA stabilization in eosinophils stimulated with TNF-α plus fibronectin [123].

Mechanisms of GM-CSF secretion have also been investigated in human lung fibroblasts [124,125,126]. Fibroblasts coculturing with macrophages is a more physiological culturing condition and GM-CSF is released from human lung fibroblasts in the presence of monocytes without agonist stimulation [124]. CREB was originally reported as a substrate of the cAMP signalling pathway. While TNF-α does not influence intracellular cAMP, TNF-α phosphorylates CREB after p38 MAPK phosphorylation in human lung fibroblasts. The p38 MAPK inhibitor, SB239063, inhibits both GM-CSF secretion and CREB phosphorylation. Cell-permeable 8-bromo-cAMP does not induce CREB phosphorylation without agonist stimulation and the PKA inhibitor, H-89 does not inhibit TNF-α-induced GM-CSF secretion, indicating that cAMP and TNF-α does not influence either CREB or the cAMP/PKA activation in human lung fibroblasts. Forskolin and phosphodiesterase-4 activate the cAMP signalling pathway by elevating intracellular cAMP. These agents inhibit TNF-α-induced GM-CSF secretion without affecting CREB phosphorylation suggesting that the cAMP/PKA pathway inhibits TNF-α-induced GM-CSF secretion distinct from CREB activity. Therefore, CREB phosphorylation is crucial for the secretion of GM-CSF after TNF-α stimulation [116]. 

**Figure 4:**
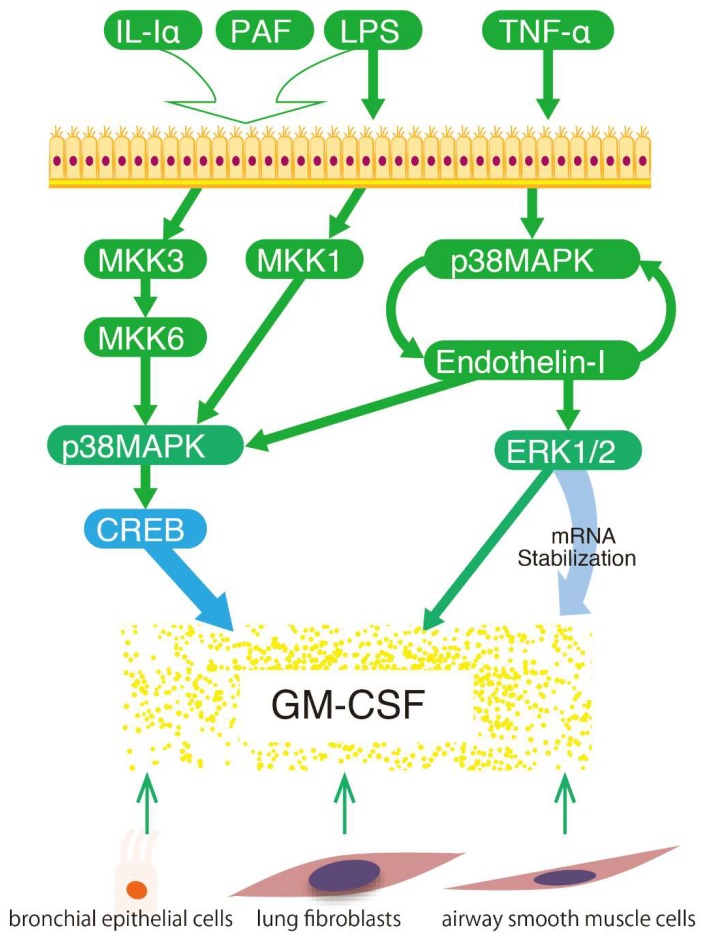
ERK1/2 and p38 MAPK-CREB-mediated GM-CSF secretion. IL-1α, PAF, LPS and TNF-α induce p38 MAPK and CREB phosphorylation. Remarkably, TNF-α induced p38 MAPK activation has a positive feedback loop mediated by endothelin-1 and facilitates both p38 MAPK and ERK1/2 activation following GM-CSF secretion and mRNA stabilization.

The significance of CREB phosphorylation in TNF-α-induced GM-CSF secretion has been demonstrated by knocking down CREB expression using three specific siRNAs against CREB in human lung fibroblasts. The p38 MAPK inhibitor SB239063 inhibited TNF-α-induced GM CSF secretion by approximately 20% and CREB siRNAs inhibited both CREB mRNA expression and GM-CSF secretion equally. These results support that p38 MAPK and CREB are critical mediators of TNF-α-induced GM-CSF secretion [116]. Recently, Gorbacheva et al. reported that the minor “G” allele of the single-nucleotide polymorphism rs928413, located in the *IL33* promoter area activates the *IL33* promoter by CREB phosphorylation via the p38 MAPK signalling pathway after TNF-α stimulation [127]. Quite recently, a positive feedback loop has been demonstrated whereby GM-CSF secreted by CREB activation stimulates CREB phosphorylation in pancreatic ductal adenocarcinoma in smokers [128]. Interestingly, Knobloch et al. reported that endothelin-1 (ET-1) also mediates a positive feedback mechanism via ERK1/2 and p38 MAPK activation in TNF-α induced GM-CSF secretion. The TNF-α-p38 MAPK cascade induced *ET-1* transcription, activates ERK1/2 and reactivates p38 MAPK in human airway smooth muscle cells. While activated ERK1/2 stimulates GM-CSF mRNA stabilization, reactivated p38 MAPK stimulates both ET-1 and GM-CSF secretion [129] (Figure 4).

## 8. Roles of ERK1/2 and p38 MAPK Mediated CREB Activation in Cytokine and Steroid Synthesis

Cytokine and chemokine production are largely dependent on transcriptional activation [130]. The previous report has shown that CREB is the main constituent of AP-1 DNA-binding activities in human neutrophils [131]. The CREB family encompasses CREB, c-AMP-1 response-element modulator (CREM) and activating transcription factor-1 (ATF-1), all of which are expressed in neutrophils. Phosphorylation of CREB at Ser133 and ATF-1 at Ser63 can be carried out by MAPKAP kinase-2 which acts immediately downstream of p38 MAPK. Phosphorylation of CREB and ATF-1 leads to increased promoter activation potential [132].

While MSK1 is known to be controlled by both the p38 MAPK and MEK/ERK module to participate in regulating their proinflammatory cytokine expression [133], the p38 MAPK-MSK1 pathway mediates proinflammatory cytokine production (CXCL8, CCL3, CCL4 and TNF-α) through LPS or TNF-α induced CREB activation in human neutrophils. CREB participates in this functional inflammatory response of neutrophils, as well as with NF-κB and C/EBP factors [76]. CREB is constitutively associated with the proximal promoter region of several chemokines such as CXCL8, CCL3 and CCL4. In addition, CREB is required for the inducible expression of CXCL8 and IL-1β in human monocytic cell lines [134,135] and for the expression of CCL4 and TNF-α in human T-cells [136] and murine macrophages [137,138]. On the other hand, ERK1/2 and p38 MAPK co-ordinately activate the MSK1/2-CREB pathway in the LPA-induced IL-8 secretion [139]. The iron chelator, deferoxamine (DFO) also triggers IL-8 secretion in human intestinal epithelial cells by phosphorylating ERK1/2 and p38 MAPK. In the process of DFO-induced IL-8 secretion, ERK1/2 activates CREB and AP-1 and p38 MAPK stabilizes IL-8 mRNA [140]. MAP kinase phosphatase-1 (MKP-1) is a phosphatase that deactivates MAPK and results in negative feedback of MAPK signalling [141]. In airway smooth muscle cells, Sphingosine 1-phosphate, a bioactive sphingolipid, elevated in asthmatic airways, upregulates MKP-1 through the protein kinase A mediated p38 MAPK-CREB pathway [142]. A recent report has shown that Ras-related small G-protein, Rit, regulates CREB function for cell survival via the p38 MAPK-MSK1/2 signalling pathway in response to various stresses [143].

Several studies have shown that the interaction between ERK1/2 and p38 MAPK is associated with steroidogenesis. Dang et al. reported that the ERK1/2 and p38 MAPK pathways activate steroidogenesis in human granulosa-lutein cells. IL-1β also activates both ERK1/2 and p38 MAPK and thus regulates steroidogenic acute regulatory protein (StAR) and progesterone synthesis [144]. Ang II and ROS activate StAR expression and steroid synthesis via p38 MAPK-CREB signalling [145,146].

cAMP induces CREB activation via ERK/p38 MAPK pathway. Forskolin-induced CREB phosphorylation is mediated by cAMP/PKA and by a time-delayed cAMP/PKA-dependent p38 MAPK/MSK1 pathway in NIH 3T3 cells. cAMP/PKA is the major pathway of CREB phosphorylation in forskolin-treated NIH 3T3 cells but PKA also phosphorylates CREB through p38 MAPK/MSK1. PKA-induced CREB activation via p38 MAPK-MSK1 is delayed compared to cAMP–PKA-induced CREB activation [147]. Upon stimulation of exenatide, glucagon-like peptide (GLP)-1 analogue, with LPS, cAMP/PKA/CREB and the cAMP/PKA/p38 MAPK/CREB signalling pathway, has been observed in cultured microglial cells. The cAMP/PKA/CREB pathway regulates exenatide-induced IL-4 secretion, while arginase 1, CD206 and IL-10 expression were mediated by the cAMP/PKA/p38 MAPK/CREB signalling pathway [148].

## 9. Roles of cAMP in CREB Activation

The roles of cAMP in CREB activation are varied depending on physiological conditions. The cAMP-PKA signalling pathway has an inhibitory effect on cardiac fibrosis. cAMP inhibits gene activation and TGFβ-Smad signalling by disrupting the interactions between Smad3 and co-activators (CREB-binding protein:CBP and its paralogue p300) in a PKA-dependent manner [149]. Treatment with forskolin and cell-permeable cAMP inhibits TGFβ and AG II-stimulated collagen synthesis, α-smooth muscle actin expression and conversion of cardiac fibroblasts to myofibroblasts [150]. Isoproterenol as the cAMP-elevating agent also inhibits the profibrotic effects of TGFβ by inhibiting ERK1/2 activation in cardiac fibroblasts [151]. Epac1 suppression in cardiac fibroblasts is occurred after myocardial infarction and Epac1 overexpression inhibits TGFβ1-induced collagen synthesis [152]. Epac1-activation reduces cardiac dysfunction and left atrial fibrosis post-myocardial infarction [153,154].

RANKL increases the adenylate cyclase 3 expression and the intracellular cAMP levels in osteoclasts. The suppression of adenylate cyclase 3 enhances osteoclastogenesis in vitro and bone resorption in vivo [155]. Wnt3a-induced NFATc1 phosphorylation is inhibited by cAMP/PKA signalling pathway. cAMP/PKA pathway inhibits osteoclast differentiation via Wnt3a-NFATc1 suppression [156]. Crosstalk between cAMP/PKA and Erk/p38 MAPK signalling pathway has opposite effects on osteoclast differentiation depending on agonist stimulation. Stimulation of adenosine, which acts at adenosine A(2A) receptors (A(2A)R), activates RANKL-induced ERK1/2 phosphorylation in a PKA-dependent manner and inhibits osteoclast differentiation mediated by inhibition of NFκB nuclear translocation [157]. In contrast, Prostaglandin E2 (PGE2) stimulates osteoclast differentiation together with RANKL [158]. The cAMP-PKA signalling pathway enhances osteoclast differentiation induced by RANKL with PGE2 stimulation. cAMP-dependent PKA phosphorylates transforming growth factor-activated kinase 1 (TAK1) at Ser412 residue in osteoclast precursors in response to PGE2 stimulation with RANKL. TAK1 synergistically enhances osteoclast differentiation by activating p38 MAPK and NF-κB in response to RANKL with PGE2 [159]. 

Forskolin also increases MUC5AC production by elevating cAMP, indicating that cAMP-PKA signalling mediates IL-1β-induced MUC5AC mucin production in NHTBE [160]. It has been reported that GLP-1 significantly inhibited ovalbumin-induced MUC5AC production, possibly through PKA-dependent inactivation of NF-κB in mice [161].

The roles of cAMP in VSMC migration are controversial [162]. cAMP-PKA signalling inhibits VSMC migration/proliferation [163]. Epac is independent of PKA activation and enhances VSMC migration [164]. A cAMP analogue selective to PKA decreases migration, whereas an Epac-selective analogue enhances migration in VSMC. Consistently, adenovirus-mediated gene transfer of PKA decreases VSMC migration, whereas that of Epac1 significantly enhances VSMC migration [165]. Epac1-deficient VSMC migration is significantly attenuated the elevation of intracellular Ca^2+^ and VSMC migration [166].

The inhibitory mechanism of GM-CSF secretion has been also investigated. Increased cAMP levels, by the treatment of phosphodiesterase 4 (PDE4) or the addition of cell-permeable cAMP, inhibits GM-CSF secretion without CREB inactivation [116]. Forskolin which is a potent cAMP-elevating agent, also inhibits TNF-α induced GM-CSF secretion. Roflumilast and Rolipram, PDE4 inhibitors used for the treatment of COPD, inhibit TNF-α-induced GM-CSF secretion in a dose-dependent manner. Roflumilast does not affect CREB phosphorylation [126]. In TNF-α induced GM-CSF secretion, CREB phosphorylation is a critical mediator, while the cAMP signalling pathway is a suppressor. A PDE4 inhibitor may be more useful for a patient with Asthma and COPD Overlap (ACO), suppressing inflammatory cytokine secretion.

Roles of cAMP and ERK/p38 MAPK pathway and crosstalk between cAMP and ERK/p38 MAPK pathway on CREB-induced physiological functions are shown in Table 1, cAMP pathway has opposite effects depending on agonist stimulation. Furthermore, the effect of cAMP-PKA pathway is different from that of cAMP-Epac pathway in VSMC migration, whereas ERK/p38 MAPK pathway upregulates CREB-induced physiological functions. Further investigations are expected to clarify the effect of crosstalk between cAMP and ERK/p38 MAPK pathway.

## 10. Conclusions

Direct phosphorylation of CREB at Ser133 by PKA and CaMKIV has been reported [14,15]. RSK and MAPKAP kinase 2 are CREB kinases mediated by ERK and p38 MAPK, respectively [167]. MSK1/2 is a common downstream kinase of ERK/p38 MAPK as a CREB kinase [75,139]. ERK1/2 and p38 MAPK have many substrates to regulate the inflammatory response and we focused on CREB in the ERK1/2 and p38 MAPK signalling pathways. ERK1/2 and p38 MAPK collaboratively regulate various physiological phenomena, underlying diseases associated with atherosclerosis, osteoporosis, airway inflammation and hormonal production. Regulatory mechanisms of CREB activation by p38 MAPK but not ERK1/2, has been investigated in periostin production, osteoclast differentiation and GM-CSF secretion. Detailed, distinct roles of ERK1/2 and p38 MAPK have become clear in bone homeostasis. Furthermore, cooperative activation of CREB by ERK1/2 and p38 MAPK has been elucidated in mucin production, VSMC migration, IL-8 secretion and steroidogenesis. MAPK inhibitors have not been utilized for the treatment of diseases yet. Overall, the CREB-mediated signalling pathway may be an essential target for developing new treatments of inflammatory, cardiovascular and bone diseases as well as cancer [168,169]. Further investigation into the crosstalk between ERK1/2 and p38 MAPK may help identify a therapeutic target among the downstream substrates and the inhibitory agent for the substrate may be a new therapeutic in diseases in which the MAPK signalling pathway is dysregulated.

## Figures and Tables

**Table 1:** Effect of cAMP, ERK/p38 MAPK and cAMP-ERK/p38 MAPK crosstalk pathway on CREB-induced physiological functions. cAMP stimulates both PKA-CREB and Epac pathway. ERK/p38 MAPK pathway phosphorylates CREB and cAMP-ERK/p38 MAPK pathway co-ordinately phosphorylates CREB. **↑** and **↓** means up-regulation and down-regulation of physiological function, respectively. N.R.; not reported.

	cAMP Pathway	ERK/p38MAPK-CREB Pathway	cAMP-ERK/p38MAPK Pathway
Neuronal system	↑	↑	↑
Cardiac fibrosis	↓	↑	N.R.
Osteoclast differentiation	↓	↑	↓ or ↑
Mucin production	↑	↑	N.R.
VSMC migration	↓(cAMP ) ↑(Epac)	↑	N.R.
GM-CSF production	↓	↑	N.R.

## Abbreviations

CREB	cyclic AMP response element-binding protein
RSK	pp90 ribosomal S6 kinase
MSK1	Mitogen- and stress-activated protein kinase 1
CaMKs	Calcium/calmodulin-dependent protein kinases
EPAC	exchange protein directly activated by cAMP
NGF	nerve growth factor
MAPKAP kinase 2	MAPK-activated protein kinase 2
VSMC	Vascular smooth muscle cell
ACM	Alcoholic cardiomyopathy
ACA	Acetaldehyde
ADH	Alcohol dehydrogenase
ALDH	Acetaldehyde dehydrogenase
ECM	Extracellular cardiac matrix
RAS	Renin–angiotensin system
Ang II	Angiotensin II
DUSPs	Dual-specificity phosphatases
NF-κB	Nuclear factor kappa B
RANKL	Receptor activator of NF-κB, ligand
NFATc1	Nuclear factor of activated T-cells, cytoplasmic 1
RACK1	Receptor for activated C kinase 1
TRAF6	TNF receptor-associated factor 6
Pl3K	Phosphatidylinositol 3-kinase
BCAP	B-cell adaptor for Pl3K
Ambn	Ameloblastin
COPD	Chronic obstructive pulmonary disease
EGFR	Epidermal growth factor receptor
TLR	Toll like receptor
AP1	Activator protein 1
SP1	Specificity protein 1
TAK1	transforming growth factor-activated kinase 1
FOXA2	Forkhead box protein A2
NADPH	Nicotinamide adenine dinucleotide phosphate
ROS	Reactive oxygen species
Fra-2	Fos-related antigen 2
DEPs	Diesel exhaust particles
HETEs	Hydroxyeicosatetraenoic acids
ET-1	Endothelin-1
PDE4	Phosphodiesterase 4
StAR	Steroidogenic acute regulatory protein
